# Structure based functional identification of an uncharacterized protein from *Coxiella burnetii* involved in adipogenesis

**DOI:** 10.1038/s41598-024-66072-3

**Published:** 2024-07-22

**Authors:** Tajul Islam Mamun, Mohammed Bourhia, Taufiq Neoaj, Shopnil Akash, Md. A. K. Azad, Md. Sarowar Hossain, Md. Masudur Rahman, Yousef A. Bin Jardan, Samir Ibenmoussa, Baye Sitotaw

**Affiliations:** 1 Department of Epidemiology and Public Health, Sylhet Agricultural University, Sylhet, 3100 Bangladesh; 2 Laboratory of Biotechnology and Natural Resources Valorization, Faculty of Sciences, Ibn Zohr University, 80060 Agadir, Morocco; 3 Department of Pharmacology and Toxicology, Sylhet Agricultural University, Sylhet, 3100 Bangladesh; 4 Department of Pharmacy, Faculty of Allied Health Sciences, Daffodil International University, Birulia, Ashulia, Dhaka, 1216 Bangladesh; 5 Department of Pathology, Sylhet Agricultural University, Sylhet, 3100 Bangladesh; 6 Department of Pharmaceutics, College of Pharmacy, King Saud University, P.O. Box 11451, Riyadh, Saudi Arabia; 7 Laboratory of Therapeutic and Organic Chemistry, Faculty of Pharmacy, University of Montpellier, 34000 Montpellier, France; 8 Department of Biology, Bahir Dar University, P.O. Box 79, Bahir Dar, Ethiopia; 9 Faculty of Pharmaceutical Science, Assam Down Town University, Guwahati, Assam India

**Keywords:** *Coxiella burnetii*, Uncharacterized protein, In silico approach, Functional annotation, Mth938 domain, Biotechnology, Drug discovery, Microbiology

## Abstract

*Coxiella burnetii*, the causative agent of Q fever, is an intracellular pathogen posing a significant global public health threat. There is a pressing need for dependable and effective treatments, alongside an urgency for further research into the molecular characterization of its genome. Within the genomic landscape of *Coxiella burnetii*, numerous hypothetical proteins remain unidentified, underscoring the necessity for in-depth study. In this study, we conducted comprehensive in silico analyses to identify and prioritize potential hypothetical protein of *Coxiella burnetii*, aiming to elucidate the structure and function of uncharacterized protein. Furthermore, we delved into the physicochemical properties, localization, and molecular dynamics and simulations, and assessed the primary, secondary, and tertiary structures employing a variety of bioinformatics tools. The in-silico analysis revealed that the uncharacterized protein contains a conserved Mth938-like domain, suggesting a role in preadipocyte differentiation and adipogenesis. Subcellular localization predictions indicated its presence in the cytoplasm, implicating a significant role in cellular processes. Virtual screening identified ligands with high binding affinities, suggesting the protein’s potential as a drug target against Q fever. Molecular dynamics simulations confirmed the stability of these complexes, indicating their therapeutic relevance. The findings provide a structural and functional overview of an uncharacterized protein from *C. burnetii*, implicating it in adipogenesis. This study underscores the power of in-silico approaches in uncovering the biological roles of uncharacterized proteins and facilitating the discovery of new therapeutic strategies. The findings provide valuable preliminary data for further investigation into the protein’s role in adipogenesis.

## Introduction

Q fever is an influenza-like zoonotic disease caused by Gram-negative obligate intracellular pathogen *Coxiella burnetii*^1^. Domesticated bovines, ovines, and caprices are the most common sources of human infections through inhalation^2^. People who work with animals may have infection through direct or indirect contact^3,4^. The potential transmission of *C. burnetii* to humans has been associated with the consumption of raw milk and other dairy products^5,6^. The infection of *C. burnetti* in humans may result in a wide range of diseases, from acute to chronic. Although the clinical signs vary based on the forms, the acute form may result in coughing, headaches, weight loss, and even death^7,8^. Coxiellosis is the name used for Q fever when it affects animals, and the disease is often asymptomatic. Cattle and camels might suffer from infertility, endrometritis, and mastitis, while sheep and goats might have abortion, stillbirth, and premature delivery^9,10^. *C. burnetti* considered the most infectious organism ever studied that is capable of spreading disease with only a single viable organism^11^. Some early studies suggest that over half of dairy animals shed *C. burnetii* in their milk and that the bacterium may live in powdered dairy products for 3 years^4,5^.

Physicians still struggle with *C. burnetii* infection because there is no international collaboration to standardize treatment and reduce clinical outcomes^12^. To combat *C. burnetii* and promote public health worldwide, more research is needed to find safe and effective therapies. However, the research on this specific biological agent has not been conducted to the same extent as other agents, resulting in inadequate information on its proteins’ structure and functional characteristics. Limited research has been conducted on the underlying structural processes that inhibit the *C. burnetii* protein^13^. About 35% of genes in the genomes of different bacterial species are in the “uncharacterized” category^14^. Hypothetical proteins (HPs) are uncharacterized protein families or domains with unclear functionality^9,15^. Annotating HPs reduces pathogen knowledge gaps by anticipating structures and biological functions^16^. Consequently, annotation of potential protein functions has gained popularity, which may help in creating prospective medicines against targeting agents and developing effective pharmacological targets^17–19^. There has been a lot of development in recent years of technology for computers that minimizes the time and expenses of functional annotation of unknown protein^20^. Furthermore, the field of bioinformatics not only enhances the reliability of studies but also enhances the efficiency of data processing^21^. Using structure- and sequence-based methodologies, in silico approaches have been successfully used to a variety of microorganisms including *Candida dubliniensis*^22^, *Clostridium tetani*^23^, and *vibrio cholera*^19^ to analyse the HPs of these diseases.

Despite its medical importance, a substantial portion of the *C. burnetii* genome remains uncharacterized, presenting a challenge for understanding the bacterium’s pathogenesis and developing effective therapeutic strategies. Among these unexplored elements are proteins whose functions and roles in cellular processes remain elusive. In this study, we employ a comprehensive in-silico approach to characterize an uncharacterized protein from *C. burnetii*, hypothesized to be involved in adipogenesis. By integrating advanced computational tools for structure prediction, domain analysis, and functional annotation, we aim to unravel the structural features and potential functional roles of this protein. Through this endeavor, we seek to shed light on the molecular basis of adipogenesis modulation by *C. burnetii* and identify novel avenues for therapeutic intervention against Q fever.

## Methods

### Sequence extraction from *Coxiella burnetii*

**Figure 1:**
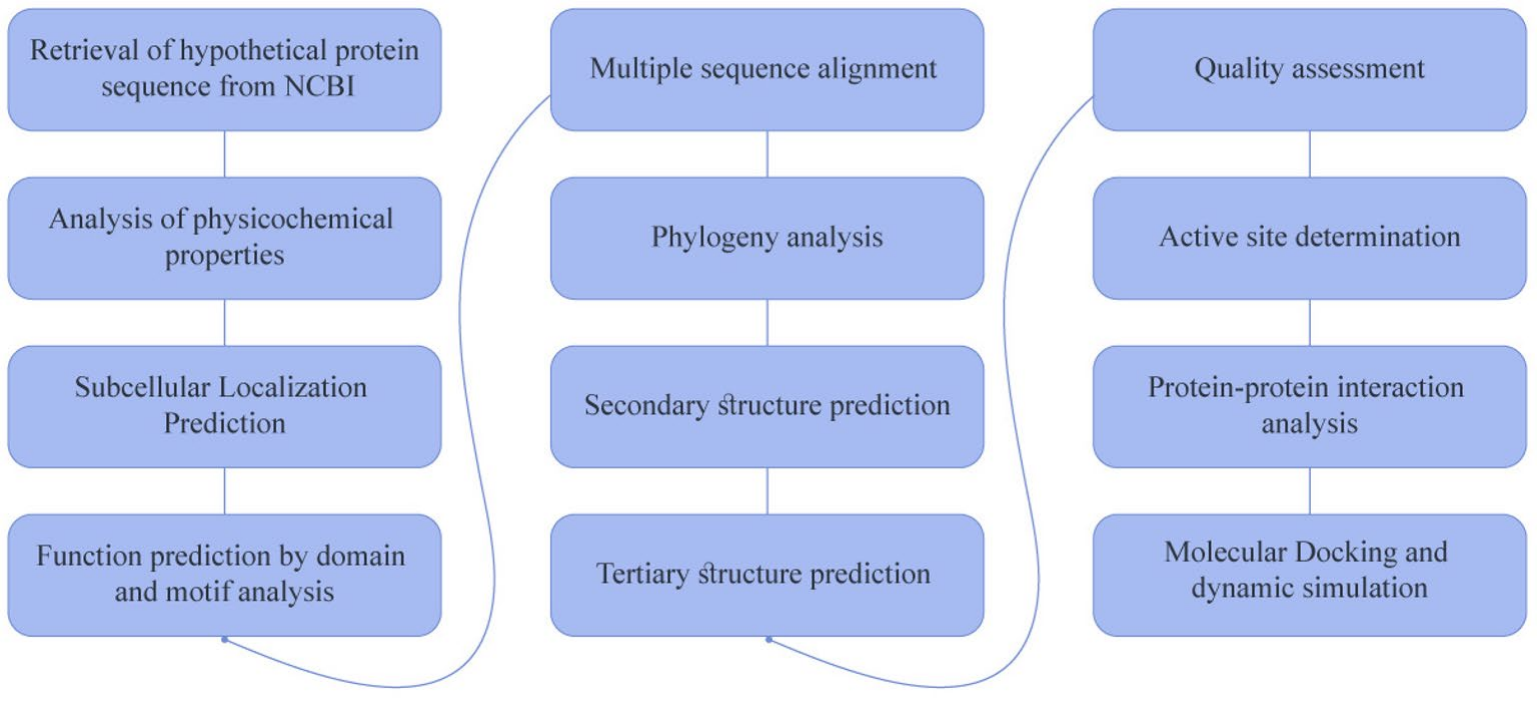
A comprehensive overview of the annotation of this study’s uncharacterized proteins.

A hypothetical or uncharacterized protein from *Coxiella burnetii* (accession no. RQM76478.1) was selected from the NCBI database^24^. The protein’s 123 amino acid residues were retained in FASTA format for further analysis. Supplementary Table 1 provides a comprehensive overview of all servers utilized to annotate the structural and functional attributes of HP. The workflow is shown in Fig. 1.

### Analysis of physicochemical properties

Protein structure and function might be better understood by exploring its physicochemical properties. The uncharacterized protein was analyzed using the ExPASy server^25^.

### Protein subcellular localization

The effective functioning of proteins and precise genetic analysis depend on subcellular localization. Drug and vaccine targets are within cells, providing significant clues for identifying and developing novel pharmaceuticals. The subcellular location of *Coxiella burnetii* was predicted using the CELLO website^26^. The findings were further verified using the PSORTb, CCTOP, SOSUIGramN, and PSLpred servers^27–30^.

### Function prediction by domain analysis

Protein functions were predicted from various database servers and tools. To identify a conserved domain and motif in the protein, the FASTA sequence was analyzed. Multiple databases and analytic tools were applied to the hypothesized protein sequence, including INTERPRO, MOTIF, Pfam, and the NCBI-hosted conserved domain database^31–34^. The MOTIF server was used to analyze protein sequence motifs. Additionally, the I-TASSER site was used to predict and document the hypothetical protein’s molecular, biological, and cellular functions^35^.

### Multiple sequence alignment and phylogeny analysis

The NCBI database of non-redundant protein sequences (nr) was searched using BLASTp with the default settings. This search resulted in the retrieval of numerous protein sequences annotated as having similar functionality. The Clustal Omega tool was used for multiple sequence alignment (MSA) to assess the query sequence against homologous sequences^36^. Finally, the tools MEGA X and BioEdit were used to conduct phylogenetic analysis and MSA^37,38^.

### Secondary structure prediction

The secondary structure of the putative protein was predicted to determine the relative placement of its amino acid residues. This analysis identified the presence of alpha-helices, beta-sheets, and random coils. To calculate the parameters of the secondary structure, we used the SOPMA tool with its default settings^39^. Finally, PSIPRED^40^ was used to further cross-check the findings from SOPMA.

### Predicting protein tertiary structure and evaluating its quality

The tertiary structure of the unknown protein was predicted to determine its arrangement and folding. The tertiary model was predicted using the Swiss Model and D-I-TASSER servers^35,41^. In the next phase of our investigation, the modeling features provided by the D-I-TASSER server were utilized. Subsequently, energy minimization was performed using the YASARA software^42^. Various web servers were used to assess the quality of the predicted protein structure. The PROCHECK web server was used to build the Ramachandran plot, which served as an indicator to evaluate the quality of the protein structure^43^. Additionally, VERIFY 3D^44^ and ERRAT^45^ servers were also used to evaluate the predicted tertiary structure of the uncharacterized protein. The Z-scores of the predicted models were computed using the ProSA server^46^.

### Active site determination

The protein active site is the precise area that enables the protein to carry out its biological functions. In the current investigation, the CASTp server was used to identify the active sites^47^. This server offers a web-based resource for identifying, delineating, and quantifying curved surface areas on three-dimensional structures of proteins. Active pockets on protein surfaces and within 3D structure interiors were precisely evaluated. Finally, PyMOL software was used to visually represent the CASTp results^48^.

### Protein–protein interaction

A computational method was applied for understanding interactions with other proteins and their significance in biological functions. The uncharacterized protein of *Coxiella burnetii* bacteria was searched in the STRING database for possible functional interaction networks^49^.

### Molecular docking analysis and dynamic simulation

Molecular docking studies were conducted between the uncharacterized protein and two distinct ligand molecules in order to further verify the conformation. In the previous literature, the ligand for the potential protein was not disclosed. Ligands were selected based on their known or predicted roles in adipogenesis or lipid-related pathways. This included compounds with reported activities as agonists or antagonists of adipogenic transcription factors, modulators of lipid metabolism enzymes, or regulators of adipocyte differentiation and function. Hence, the Protein Data Bank (PDB) server was implemented to identify potential ligands. Therefore, to investigate the interaction between the protein and potential inhibitors, docking studies were conducted using two distinct ligands: 2-[(2-phenylphenyl)amino]benzoic acid and 2-amino-4-phenylbenzoic acid. These compounds were chosen based on their structural properties, which suggest potential for high affinity binding to the target protein. This approach helps in understanding the ligands’ positions, orientations, and conformations within the binding site of the HP, thus offering valuable information for the design and optimization of more effective therapeutic agents targeting this protein.

The ligands used in the docking investigation were obtained from the PubChem server and then converted to the PDB format using PyMOL software^50^. Docking analysis was performed using AutoDock Vina through PyRx tool to predict how ligands interact with macromolecules^51^. The outcomes of the docking process were assessed using PyMOL and Discovery Studio software^52^. The Chimaera tool was used to better understand the hydrophobic and hydrogen-bonding interactions occurring between the atoms of the receptor and ligand at a proximity of 5 Å ^53^.

The mobility of molecules and atoms in dynamic systems like protein–ligand complexes are studied using molecular dynamic simulation (MDS) to explain crucial physicochemical processes. MDS were conducted on receptor-ligand complexes from molecular docking studies to confirm interaction mechanisms and stability in a dynamic environment. Normal mode analysis (NMA) on the iMODS website was implemented to determine the internal coordinates’ motion by applying MDS to the HP-ligand complexes^54^. This software can compute the deformability, B-factors, eigenvalues, covariance, and elastic network^55^.

## Result and discussion

### Protein  sequence retrieval and similarity analysis

The hypothetical protein (accession no. RQM76478.1) was randomly selected from the NCBI server. It originates from the gram-negative organism *Coxiella burnetii* and comprises 123 amino acid residues. To facilitate further investigation, the protein sequence was obtained in FASTA format. The BLASTp program was then used to search the non-redundant protein sequences (nr) database for proteins structurally similar to the chosen hypothetical protein (Table 1). According to the BLASTp result, the uncharacterized protein shows similarities with several proteins, including Mth938-like domain-containing proteins.

**Table 1:** Proteins with identical features were obtained from the non-redundant (nr) database.

Description	Scientific name	Query cover (%)	Percent identity (%)	E value	Accession
Mth938-like domain-containing protein	*Coxiella burnetii*	100	100.0	1e−85	WP_010957854.1
Mth938-like domain-containing protein	*Coxiella burnetii*	100	99.19	9e−85	WP_005768829.1
Mth938-like domain-containing protein	*Coxiella burnetii*	100	99.19	2e−84	WP_042527765.1
Mth938-like domain-containing protein	*Coxiella burnetii*	100	96.56	2e−83	WP_011996831.1
Mth938-like domain-containing protein	*Coxiella* like endosymbiont of *Ornithodoros maritimus*	100	93.50	4e−79	WP_267256646.1
Mth938-like domain-containing protein	*Coxiella* like endosymbiont of *Ornithodoros amblus*	100	90.24	1e−76	WP_280123615.1
Putative membrane spanning protein	*Coxiella mudrowiae*	100	62.60	7e−54	AKQ33729.1
Mth938-like domain-containing protein	*Coxiella mudrowiae*	100	62.60	1e−53	WP_100623104.1
Mth938-like domain-containing protein	*Coxiella* like endosymbiont of *Rhipicephalus microplus*	100	62.60	1e−53	WP_102157048.1
Mth938-like domain-containing protein	*Coxiella* like endosymbiont of* Ornithodoros maritimus*	97	60.00	2e−48	WP_264435398.1

### Analysis of physicochemical properties

The hypothetical protein’s physical and chemical characteristics were identified via ExPASy ProtParam server. The amino acid composition was Ala (7.3%), Arg (4.9%), Asn (3.3%), Asp (3.3%), Cys (1.6%), Gln (1.6%), Glu (7.3%), Gly (8.9%), His (3.3%), Ile(6.5%), Leu (11.4%), Lys (3.3%), Met (2.4%), Phe (1.6%), Pro (5.7%), Ser (7.3%), Thr (7.3%), Trp (1.6%), Tyr (4.1%), and Val (7.3%). The protein’s atomic composition includes carbon (601, 31.7%), hydrogen (951, 50.1%), nitrogen (161, 8.5%), oxygen (179, 9.4%), and sulfur (5, 0.3%). The theoretical pI of the protein was calculated to be 5.74, and its molecular weight was 13,456.43 Dalton, and GRAVY, an indicator of its hydrophobicity, was 0.002 (Table 2). It was suggested that the unknown protein was hydrophobic based on the positive GRAVY score^56^. Which indicates a lack of interaction possibilities with water. The protein was anticipated to be unstable with an instability index (II) of 57.75. A protein is considered to possess stability when its instability index value falls below 40, whereas a protein is classified as unstable if its instability index value exceeds 40^57^. The protein’s pI was determined to be 5.74, making it acidic (pH 7) in composition. The predicted protein half-life in mammalian reticulocytes in vitro is 30 h; in yeast and *E. coli* in vivo, it is > 20 h and > 10 h, respectively. These approaches may adjust half-life and adapt to treatment methods and disease^58^. The extinction coefficient stands in for a proportionality constant in the Beer-Lambert formula. It provides an approximation of the quantity of light at a certain wavelength absorbed by proteins^59^.

**Table 2:** Physicochemical characteristics of the uncharacterized protein.

Number of amino acids	123
Molecular weight	13,456.43
Theoretical pI	5.74
Total number of negatively charged residues (Asp + Glu)	13
Total number of positively charged residues (Arg + Lys)	10
Formula	C_601_H_951_N_161_O_179_S_5_
Instability index (II)	57.75
Aliphatic index	98.29
Grand average of hydropathicity (GRAVY)	0.002
The estimated of half-life	30 h (mammalian reticulocytes, in vitro) > 20 h (yeast, in vivo) > 10 h (Escherichia coli, in vivo)
Extinction coefficients (M–1 cm^−1^)	18,575 1.380, assuming all pairs of Cys residues form cystines; 18,450 1.371, assuming all Cys residues are reduced

### Estimating the subcellular location of protein

Determining subcellular localization is crucial for understanding protein function and genome analysis. Essentially, a protein’s subcellular location indicates its position within a cell, which could be the outer membrane, inner membrane, periplasm, extracellular space, or cytoplasm^60^. According to the CELLO server, the selected hypothetical protein is localized in the cytoplasm, with a reliability score of 3.301. This result was confirmed by PSORTb v3.0.3, SOSUIGramN, and PSLpred websites. Additionally, the PSLpred tool predicted a cytoplasmic localization for the protein with a score of 98.1% and a reliability index of 5. However, the CCTOP server predicts that the protein is not a transmembrane protein. Predicting the subcellular localization of an unidentified protein is valuable for genomic identification and drug development^61^. Membrane proteins may serve as vaccine targets, while cytoplasmic proteins often function as drug targets^62^. This suggests that the uncharacterized protein could be a promising candidate for drug targeting.

### Protein domain analysis for predicting biological function

**Figure 2:**
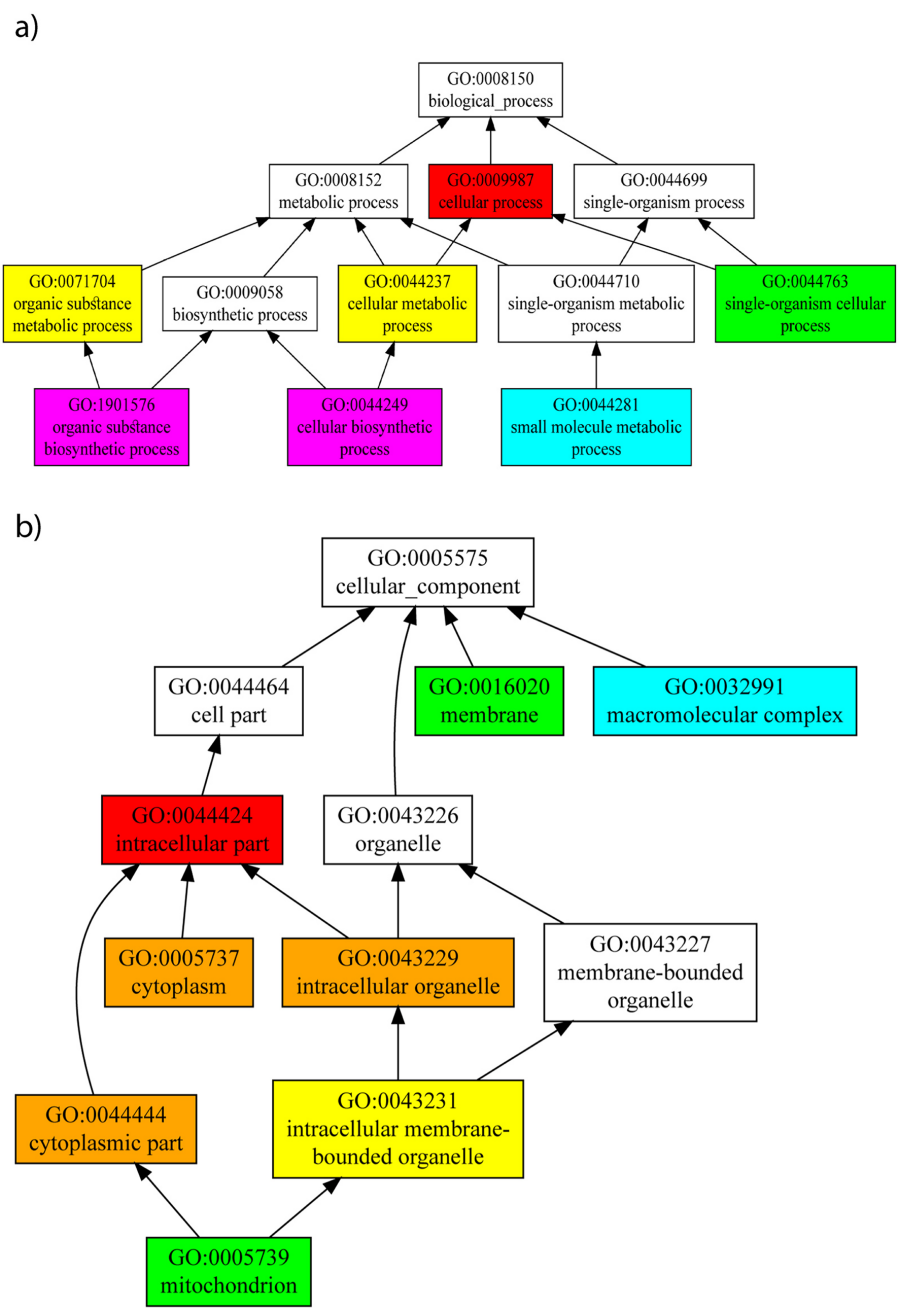
The graph shows the predicted terms within the Gene Ontology hierarchy for biological processes (**a**) and cellular components (**b**) of the selected protein. CscoreGO is the confidence score of the predicted Gene Ontology (GO) terms. CscoreGO values range from 0 to 1, where a higher value indicates greater confidence in predicting the function using the template.

According to NCBI’s CD-search, the selected hypothetical protein contains only a single domain. The Mth938-like domain-containing protein was identified as the origin of this domain (accession No. cd05560). The server generated an e-value of 3.78e-51, indicating that amino acid residues 13–121 constitute the domain. This finding was corroborated by Pfam, D-I-TASSER, and INTERPRO servers to ensure its accuracy. While INTERPRO and MOTIF attempted to identify relevant motifs, they did not yield any results. The genome of the microorganism *Methanobacterium thermoautotrophicum* (Mth) encodes a potential protein known as Mth938. According to various servers, the Mth938 domain-containing protein has the potential to be involved in both preadipocyte differentiation and adipocyte formation. Additionally, the Mth938 protein is associated with functions such as 3-oxoacyl-[acyl-carrier-protein] synthase activity, fatty acid biosynthesis, cell wall macromolecule biosynthesis, response to oxygen levels, Actinobacterium-type cell wall biogenesis, mycolic acid metabolism, ketone biosynthesis, and phenylpropanoid biosynthesis. The utilization of the D-I-TASSER server for predicting the structure and function of an uncharacterized protein from *Coxiella burnetii* has provided valuable insights into its potential role in adipogenesis. D-I-TASSER’s predictions span across molecular function, biological processes, and cellular components, offering a comprehensive view of the protein’s role within the cellular environment (Fig. 2). D-I-TASSER suggests that the protein exhibits binding activities, possibly relating to lipid or receptor molecules, which is consistent with mechanisms known to influence adipocyte differentiation and function. This insight supports the hypothesis that the protein may directly interact with key regulators of adipogenesis. The tool predicts involvement in signaling pathways and cellular processes that are crucial for adipocyte differentiation. Such processes may include the regulation of transcription factors or activation of enzymes involved in lipid metabolism, highlighting a potential mechanism through which the protein influences adipogenesis. The protein is predicted to be associated with cellular locations that are integral to adipocyte function, such as the endoplasmic reticulum or lipid droplets. This localization supports its proposed role in adipogenesis and suggests a direct pathway through which the protein may exert its effects.

In aggressive estrogen receptor-positive breast cancers, the uncharacterized oncogene adipogenesis associated Mth938 domain containing (AAMDC) regulates metabolic enzymes involved in the one-carbon folate and methionine cycles, as well as lipid metabolism^63^. A preliminary study suggests that the post-transcriptional regulation of AAMDC occurs through alternative polyadenylation (APA) and microRNAs^64^. During adipogenesis, AAMDC might activate the caspase-3 pathway to turn on the preadipocytes from proliferation to differentiation and to keep it viable^65^.

Studies of conserved domains show that the C11orf67 gene of human seems to fold very similarly to a predicted protein from the bacterium *Methanobacterium thermoautotrophicum* (Mth938)^66^. Furthermore, it was determined that C11orf67 demonstrated overexpression in several breast cancer cell lines^67^. No previous research has been conducted on Mth938 domain of the *Coxiella burnetii* bacterium. Nevertheless, there is some structural similarity between human C11orf67 and Mth938 domain of *Coxiella burnetii*. The expression of Mth938 domain, which is crucial for several biological processes, including preadipocyte differentiation and adipogenesis, has to be strictly monitored. Drawing on literature that describes the involvement of Mth938 domain-containing proteins in cellular processes relevant to adipocyte differentiation and lipid metabolism, we hypothesize potential mechanisms through which our protein might impact adipogenesis. This includes potential interactions with key adipogenic regulators and involvement in signaling pathways that drive adipocyte maturation and function. We also expanded our discussion to consider how the protein’s function might relate to the biology of *Coxiella burnetii*, especially given its intracellular lifestyle and interaction with host lipid metabolism. This aspect is particularly intriguing as it offers a dual perspective on the protein’s role: its contribution to the pathogen’s survival and replication within host cells, and its potential impact on host cellular processes, such as adipogenesis.

### Alignment of sequences and analysis of phylogenetic relationships

**Figure 3:**
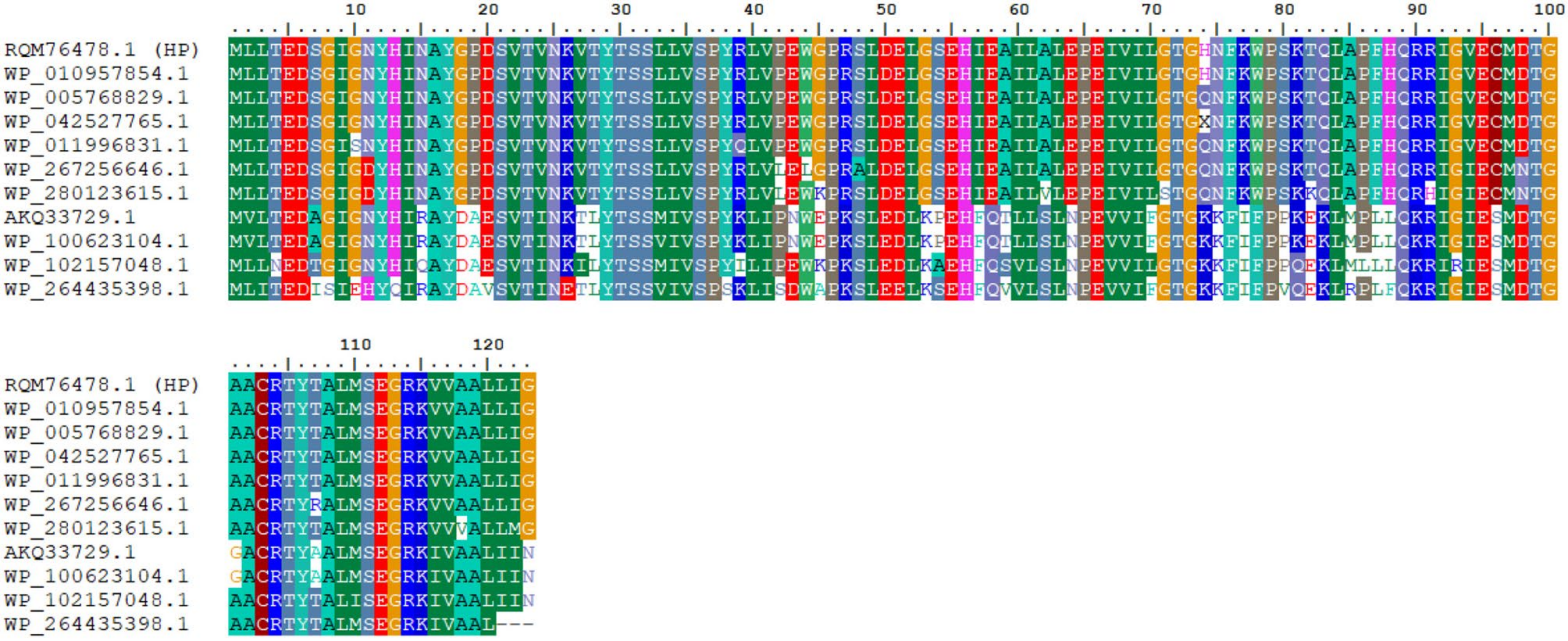
Multiple sequence alignments of different Mth938-containing proteins were conducted using BioEdit software, revealing conserved regions across the sequences.

BLASTp was performed on the NCBI protein database against a non-redundant protein sequence database, revealing similarities between the target protein and various proteins, particularly those containing Mth938-like domains. For multiple sequence alignment (MSA) and phylogenetic analysis, organisms with the most similar protein sequences, lowest e-values, and highest query coverage were selected (Table 1). Subsequently, MSA was conducted using BioEdit software, yielding consistent results with both MUSCLE and Clustal Omega servers (Fig. 3). The MSA analysis revealed a significant level of conservation among amino acid residues in the target protein.

A phylogenetic tree was generated using the MEGA 11 software, employing 1000 bootstrap replications and the neighbor-joining technique (Supplementary Fig. 1). Phylogenetic analysis determined the evolutionary distance between the target protein and aligned similar proteins, confirming their similarity. The tree root represents the ancestral lineage, while branch points represent descendants or proteins of the ancestor. A phylogeny illustrates common ancestry among lineages, with each lineage having distinct common ancestry. Proteins in the top five branches share a more recent common ancestor than those in the bottom half of the tree. Closer proximity on the phylogenetic tree indicates a more recent common ancestor among proteins.

### Prediction of protein secondary structure

**Figure 4:**
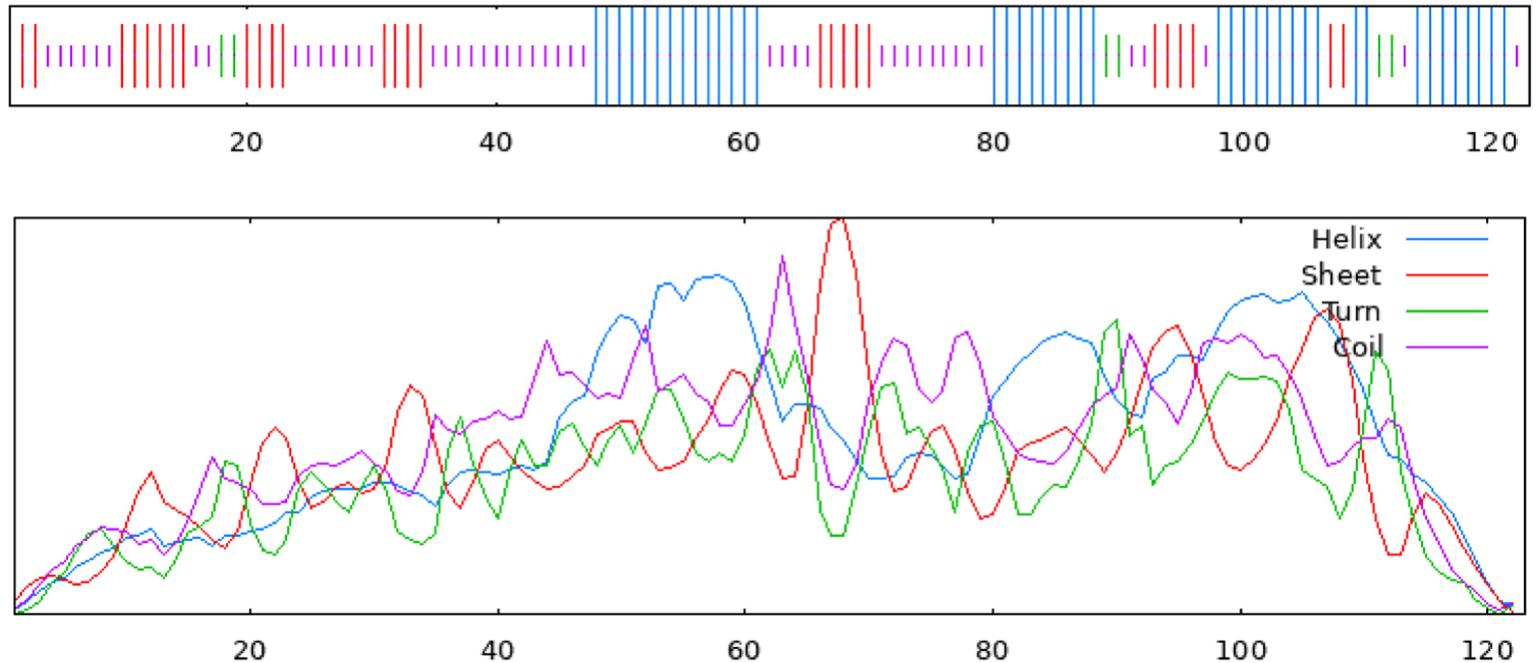
The SOPMA server provides a visualization of the predicted secondary structure, offering insights into the helices, sheets, and coils expected within the protein based on its amino acid sequence.

According to the SOMPA server, the hypothetical protein’s secondary structure was composed mostly of random coils (37.40%), alpha helices (34.15%), extended strands (23.58%), and beta turns (4.88%) (Supplementary Fig. 2). The data were compared using PSIPRED, and the results were found to be consistent (Fig. 4). Secondary structure analysis is crucial for understanding protein function, as there is a close relationship between protein structure and function. Predicting protein secondary structure can also aid in forecasting tertiary structure, establishing a connection between the primary sequence and the tertiary structure^68^.

### Predicting tertiary structures and assessing quality

**Figure 5:**
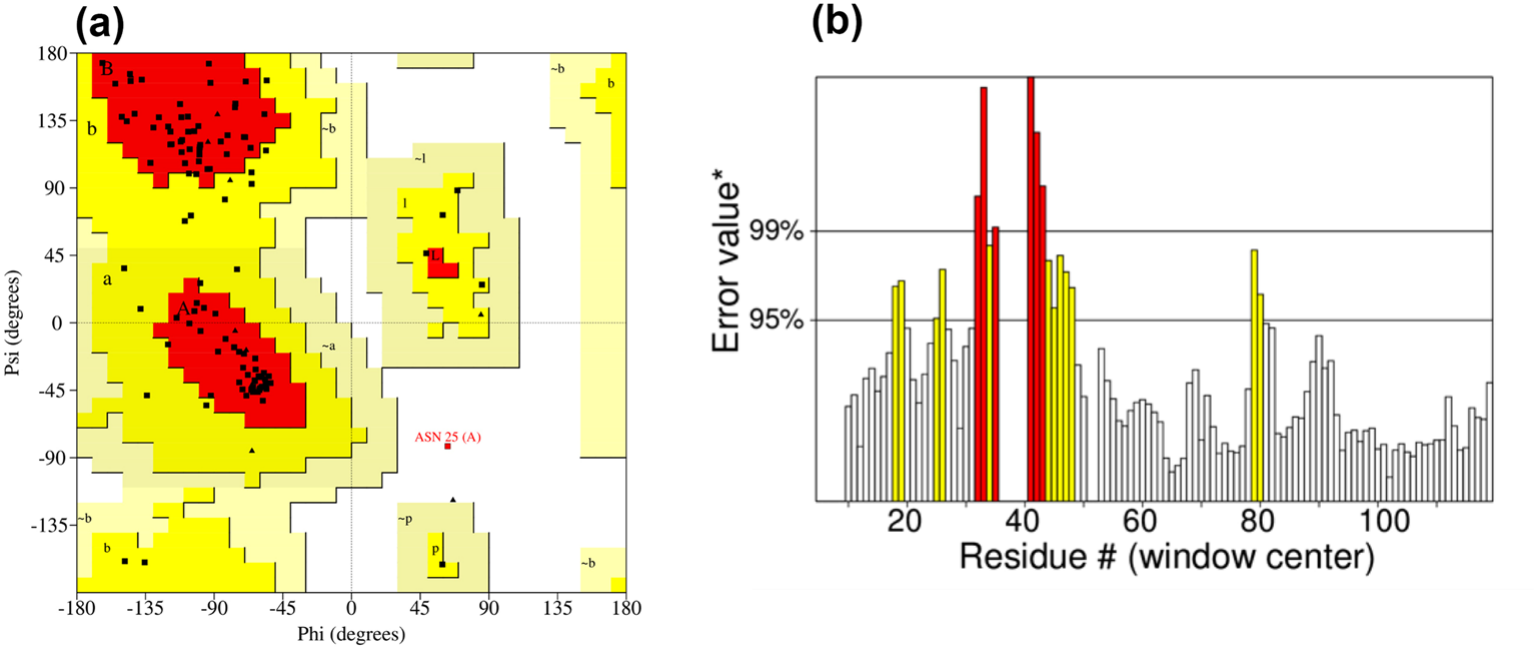
Quality assessment of the predicted structure includes: (**a**) a Ramachandran plot for the constructed frame, validated by the PROCHECK server, and (**b**) the overall quality factor of the target model as evaluated by the ERRAT server.

**Figure 6:**
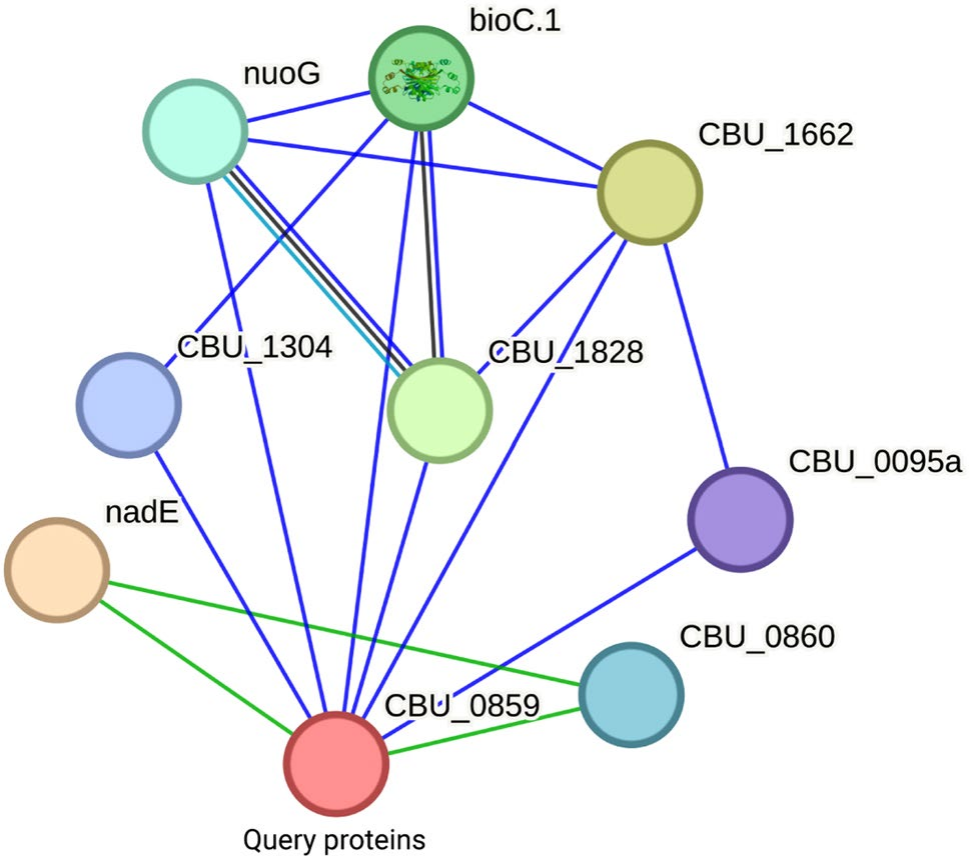
The interactions of the target hypothetical protein with other proteins are depicted in a STRING network analysis. Unknown proteins are represented by empty nodes, while proteins with known or expected 3D structures are represented by full nodes.

The *Coxiella burnetii* protein tertiary structure was anticipated through D-I-TASSER, and Swiss Model websites. The Swiss Model server predicted that the protein’s 3D structure would share 37.40% identity with the top-scoring template (PDB ID: 2 gm2.1. A). Additionally, the oligo-state of the protein was found to be a monomer by additional verification of the 3D structure prediction using SWISS-MODEL interactive workspace^69^. Following the generation of 3D structures from the D-I-TASSER server, the protein’s structure was verified through multiple quality assessment stages and use for further analysis (Supplementary Fig. 3). The PROCHECK software used Ramachandran plot analysis to evaluate the 3D model (Table 3; Fig. 5). According to the results given by the Ramachandran Plot, 80.8% of the residues were found in the most favorable locations. In the most favorable locations, it is commonly accepted that more than 90% of the residues will provide a reliable 3D model^70^. After that, VERIFY 3D and ERRAT were used to double-check the results; and it was found that 52.03% of the residues had an average 3D-1D score >  = 0.1. The YASARA energy minimization server subsequently modified the 3D structure. After energy reduction, − 39,228.4 kJ/mol becomes − 61,161.1 kJ/mol. The initial score was − 2.40, and the energy minimization was enhanced to − 0.73. Figure 6 shows that after energy reduction, ERRAT evaluated the model as having a high-quality factor of 84.07% compared to its initial value of 82.52%. A high-quality model is defined as one with a score greater than 50; higher scores indicating a better-quality model^71^. The anticipated structure’s quality factors pre and post energy minimization are given in Table 4. Subsequently, the predicted structure was evaluated using the ProSA-web tool, which revealed a Z score value of -6.79 (Supplementary Fig. 4).

**Table 3:** Ramachandran plot statistics for the predicted 3D model of the uncharacterized protein.

Ramachandran plot analysis	No. (%)
Residues in the most favored regions [A, B, L]	84 (80.8%)
Residues in the additional allowed regions [a, b, l, p]	19 (18.3%)
Residues in the generously allowed regions [− a, − b, − l, − *p*]	0 (0.0%)
Residues in the disallowed regions	1 (1%)
No. of non-glycine and non-proline residues	104 (100%)
No. of end-residues (excl. Gly and Pro)	1
No. of glycine residues (shown in triangles)	11
No. of proline residues	17
Total no. of residues	123

**Table 4:** Comparison of the quality assessment of the protein before and after energy minimization.

Criteria	Pre-energy- minimization	post-energy- minimization
Energy	− 39,228.4 kJ/mol	− 61,161.1 kJ/mol
Ramachandran plot (PROCHECK)	80.8%	87.5%
Quality factor (ERRAT)	82.52%	84.07%
VERIFY 3D	52.03% of the residues had an average 3D–1D score > = 0.1	65.85% of the residues had an average 3D–1D score > = 0.1

### Determination of the active site

The CASTp server calculated putative protein active sites using a probe radius of 1.4 Å to determine solvent-accessible surface area. The active pockets’ volumes, areas, and mouth openings were also measured. According to the findings, the protein had 24 active pockets, with the optimal active site having a surface area of 375.367 and a volume surface area of 363.460. Analytical calculations were made using both the Lee and Richards’ surface^72^ model for solvent accessibility and Connolly’s surface model for molecule surfaces^73^. Supplementary Fig. 5 illustrates the active sites of interactions. Accurately localizing the active site on a protein holds significant importance in the fields of molecular docking and the development of novel pharmaceuticals and vaccines.

### Protein–protein interaction analysis

The STRING server provides comprehensive coverage of both direct and indirect associations among anticipated protein–protein interactions (PPIs). The protein of interest has connections to other proteins, according to protein–protein interaction analysis, some of which have been experimentally annotated with known functions and others that have not (Fig. 6). The target protein is anticipated to interact with nadE in a significant way. The identified anticipated interaction partners and their scores are nadE (0.773), bioC.1 (0.698), nuoG (0.592), CBU_1304 (0.509), and CBU_0095a (0.504). Furthermore, several proteins have engaged in interactions with some unidentified proteins whose functional characteristics have not yet been characterized. Among these, nadE is the most important because it is a glutamine-dependent NAD(+) synthetase that also accelerates the ATP-dependent amidation of deamido-NAD to produce NAD. L-glutamine is used as a source of nitrogen. BioC.1 and CBU_1304 are involved in synthesizing pimeloyl-ACP via the fatty acid synthetic pathway and the oligoketide cyclase/lipid transport protein. The mechanisms of disease processes may be better understood with the use of protein–protein interaction data^74^.

### Molecular docking  and dynamic simulation

**Figure 7:**
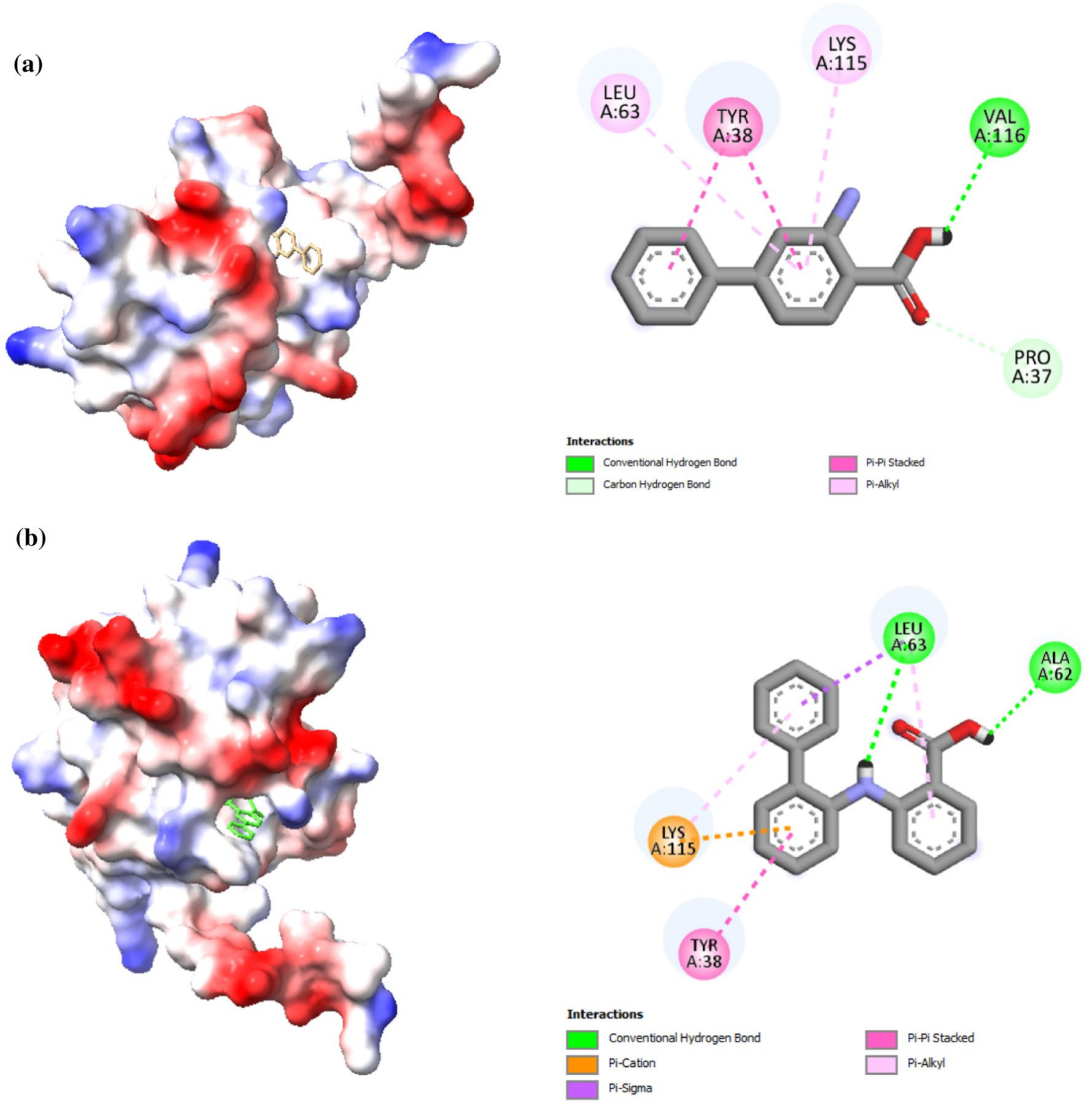
The intermolecular interactions between the target protein (HP) and probable ligands are illustrated as follows: (**a**) Interaction between the targeted protein and the ligand 2-Amino-4-phenylbenzoic acid, and (**b**) Interaction between the targeted protein and the ligand 2-[(2-phenylphenyl)amino]benzoic acid.

The molecular docking method exemplifies a structure-based drug design approach that mimics molecular interactions and predicts binding mechanisms between receptors and ligands^75^. Consequently, in order to explore the interactions between the host protein and prospective inhibitors, docking analyses were carried out with two different ligands: 2-[(2-phenylphenyl)amino]benzoic acid and 2-amino-4-phenylbenzoic acid. The docking study incorporating the hypothetical protein and the ligand was carried out with the help of Autodock Vina software. The blind docking procedure was conducted in order to examine the specificity of the substrates. Afterwards, the software PyMOL and Discovery Studio were employed to do further investigation of the interactions subsequent to the docking study. The MD analysis revealed many intermolecular interactions between the protein and probable ligands. 2-[(2-phenylphenyl)amino]benzoic acid had a high binding affinity for the protein, with a docking score of − 7.00 (kcal/mol). A binding affinity of − 6.20 (kcal/mol) was observed for 2-amino-4-phenylbenzoic acid through docking (Fig. 7). The compounds exhibiting the lowest docking score were considered excellent and demonstrated a higher level of affinity^76^. This approach helps in understanding the ligands’ positions, orientations, and conformations within the binding site of the HP, thus offering valuable information for the design and optimization of more effective therapeutic agents targeting this protein.

Blind docking shows that 4 and 5 amino acid residues of the HP interact with the docked complexes of HP-2-[(2-phenylphenyl)amino]benzoic acid and HP-2-Amino-4-phenylbenzoic acid. The study’s active site residue-ligand interaction helps us understand residue-drug binding mechanisms. It obtained substantial discoveries about biological structure and function and hydrogen bonding. The complexes of HP-2-[(2-phenylphenyl)amino]benzoic acid have 2 conventional hydrogen bonds. HP-2-amino-4-phenylbenzoic acid only possessed a single conventional hydrogen bond. The ligands of 2-[(2-phenylphenyl)amino]benzoic acid interact with the residues Glu63, Leu63, Ala62, Ly115, and Tyr38, and the 2-Amino-4-phenylbenzoic acid ligands interact with the residues Leu63, Tyr38, Lys115, Val116, and Pro37. Both ligands had similar interaction residues, including Leu63, Lys115, and Tyr38, in their structures. The molecules with a lower docking value were thought to be the best and had a greater degree of affinity (Table 5).

**Table 5:** The summary provides insights into the extent and specificity of protein binding interactions involving *Coxiella burnetii*, shedding light on the affinity levels.

Ligand	Binding affinity (kcal/mol)	RMSD (Å)	Interacting residues
2-Amino-4-phenylbenzoic acid	− 6.20	0.00	Leu63, Tyr38, Lys115, Val116, Pro37
2-[(2-phenylphenyl)amino]benzoic acid	− 7.00	0.00	Leu63, Ala62, Lys115, Tyr38

**Figure 8:**
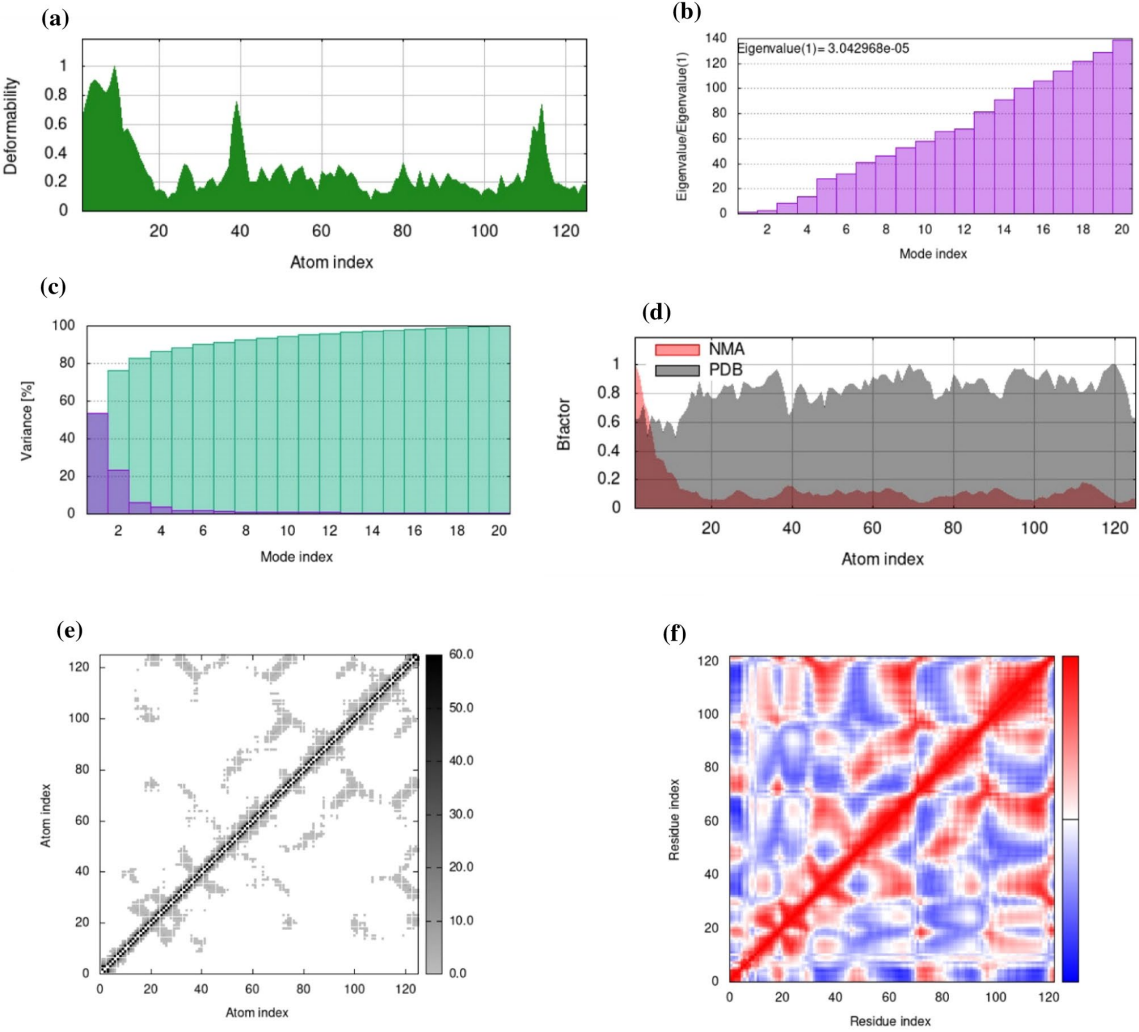
Molecular dynamics simulation was conducted for HP in complex with 2-[(2-phenylphenyl)amino]benzoic acid, analyzing various parameters: (**a**) deformability (**b**) eigenvalue (**c**) variance (**d**) B factor (**e**) elastic network (**f**) covariance matrix.

Normal mode analysis (NMA) on the iMODS server was used to measure the effectiveness and stability of the complexes. We selected HP-2-[(2-phenylphenyl)amino]benzoic for the molecular dynamic simulation investigation because of its high binding affinity and evaluated the atomic dynamic motions inside the complexes. NMA B-factor values, which are proportional to the RMS value, reflect the docked complex’s mobility. The complex’s deformability relies on the residues’ distortions, embodied in chain hinges. The main-chain deformability is a measure of the capability of a given molecule to deform at each of its residues. The location of the chain ‘hinges’ can be derived from high deformability regions (Fig. 8a). The correlation between the experimental B-factor from crystallography or NMR data and the calculated B-factor from Normal Mode Analysis (NMA) is a crucial aspect of structural biology, particularly in understanding protein dynamics. The calculated from NMA is obtained by multiplying the NMA mobility by 8pi^2^L^. Actually, the B-factor column gives an averaged RMS (Fig. 8d).

. The correlation between experimental and calculated B-factors serves as a validation of the NMA model. A high correlation suggests that the NMA successfully captures the essential motions contributing to the protein’s dynamic behavior as observed experimentally. Conversely, discrepancies might indicate areas where the model fails to account for certain movements or where the experimental data might be limited or averaged (as in the case of NMR-derived B-factors). The eigenvalue obtained for your docked complex, 3.0429e−05, suggests that the complex has a relatively low stiffness along the corresponding mode of motion (Fig. 8b). In practical terms, this means that the complex is more prone to deformation or conformational changes along this particular mode compared to complexes with higher eigenvalues. Understanding the stiffness properties of protein complexes is crucial in drug design and therapeutics. Targeting protein–protein interactions with small molecules or peptides often involves disrupting specific modes of motion within the complex. For each normal mode, there existed a correlation between eigenvalue and variance^77^. The covariance diagram describes the motion of a particular region of the molecule, with red, blue, and white colors representing movements that are linked, unrelated, and anti-related, accordingly (Fig. 8f). On the iMODS server, this correlation matrix is used to derive normal modes in a simplified manner, facilitating the exploration of possible collective motions under physiological conditions with minimal computational resources. The server utilizes the correlation matrix to identify patterns of concerted atomic fluctuations, indicating regions of the protein that move together in a correlated manner. The relationship between the variance associated with each normal mode and the eigenvalue is inversely related, meaning that higher eigenvalues correspond to lower variances and vice versa (Fig. 8c). This relationship is crucial for understanding the distribution of motions within a protein structure as described by normal mode analysis (NMA). In visual representations of NMA results, colored bars are commonly used to depict the individual and cumulative variances associated with each normal mode. Modes with higher variances contribute more significantly to the overall flexibility of the protein and may represent functionally relevant motions, such as conformational changes associated with ligand binding or enzyme catalysis. The graph displays the relative contribution of variance to equilibrium movements for each normal mode, with green and violate bars representing group and single variance. In addition, a model of an elastic network was constructed, which could identify spring-connected atomic pairs. The elastic network model defines which pairs of atoms are connected by springs. Each dot in the graph represents one spring between the corresponding pair of atoms. Dots are colored according to their stiffness; the darker grays indicate stiffer springs and vice versa. When visualizing the ENM using a graph representation, each dot in the graph represents a spring connecting a pair of atoms. The color of the dots corresponds to the stiffness of the springs they represent. Typically, darker shades of gray indicate stiffer springs, while lighter shades indicate fewer rigid connections (Fig. 8e).

## Conclusion

In this study, we conducted a comprehensive in-silico analysis of an uncharacterized protein from *Coxiella burnetii*, unveiling insights into its structural and functional attributes. Our findings suggest its involvement in vital biological processes, such as preadipocyte differentiation and adipogenesis, inferred from the presence of a conserved Mth938-like domain. The protein’s localization in the cytoplasm underscores its significance in cellular function. Through computational analyses, we identified potential ligands with strong binding affinities, highlighting the protein’s potential as a promising drug target for Q fever and related diseases. Molecular dynamics simulations supported the stability of the protein-ligand complexes, reinforcing their therapeutic relevance. Further experimental validation is necessary to confirm the protein’s functions and interactions, providing potential avenues for the development of novel therapies. Overall, this study contributes to bridging the knowledge gap regarding the biological roles of uncharacterized proteins in *C. burnetii.*

### Limitations

We acknowledge several limitations inherent to our study. First, despite the advancements in computational methods, such as D-I-TASSER for protein structure prediction and functional annotation, these in-silico predictions require experimental validation to confirm the hypothesized functions and interactions. The accuracy of these computational models can be constrained by the quality and extent of existing structural and functional databases, which may not fully represent the diversity of protein structures and functions in nature. Furthermore, the specific environmental conditions within a host organism, such as the presence of other interacting proteins, post-translational modifications, and local cellular conditions, can significantly influence protein function. These factors are challenging to fully replicate or account for in computational models. Consequently, our predictions, while informative, may not capture the full complexity of the protein’s role in adipogenesis as it occurs in vivo. Additionally, our focus on *Coxiella burnetii* and its potential implications in adipogenesis may overlook broader interactions and functions relevant to the organism’s lifecycle or pathogenicity. The role of the identified protein within the context of the bacterium’s intracellular environment and its interactions with host cells might also reveal further dimensions of its function that our current approach cannot elucidate. In conclusion, while our study employs state-of-the-art in-silico techniques to uncover potential functions of an uncharacterized protein, it is crucial to approach these findings as hypotheses that lay the groundwork for future experimental investigations. Validating these predictions through laboratory experiments is essential for confirming their accuracy and for understanding the protein’s role in the context of *Coxiella burnetii’*s biology and its interaction with host organisms.

## Supplementary Information

Supplementary Information.

## Data Availability

All data generated or analysed during this study are included in this published article.
