# Author Correction: Structure based functional identification of an uncharacterized protein from *Coxiella burnetii* involved in adipogenesis

**DOI:** 10.1038/s41598-024-70969-4

**Published:** 2024-09-10

**Authors:** Tajul Islam Mamun, Mohammed Bourhia, Taufiq Neoaj, Shopnil Akash, Md. A. K. Azad, Md. Sarowar Hossain, Md. Masudur Rahman, Yousef A. Bin Jardan, Samir Ibenmoussa, Baye Sitotaw

**Affiliations:** 1 Department of Epidemiology and Public Health, Sylhet Agricultural University, Sylhet, 3100 Bangladesh; 2 Laboratory of Biotechnology and Natural Resources Valorization, Faculty of Sciences, Ibn Zohr University, 80060 Agadir, Morocco; 3 Department of Pharmacology and Toxicology, Sylhet Agricultural University, Sylhet, 3100 Bangladesh; 4 Department of Pharmacy, Faculty of Allied Health Sciences, Daffodil International University, Birulia, Ashulia, Dhaka, 1216 Bangladesh; 5 Department of Pathology, Sylhet Agricultural University, Sylhet, 3100 Bangladesh; 6 Department of Pharmaceutics, College of Pharmacy, King Saud University, P.O. Box 11451, Riyadh, Saudi Arabia; 7 Laboratory of Therapeutic and Organic Chemistry, Faculty of Pharmacy, University of Montpellier, 34000 Montpellier, France; 8 Department of Biology, Bahir Dar University, P.O. Box 79, Bahir Dar, Ethiopia; 9 Faculty of Pharmaceutical Science, Assam Down Town University, Guwahati, Assam India

Correction to: *Scientific Reports* 10.1038/s41598-024-66072-3, published online 22 July 2024

The original version of this Article contained an error in the grant number listed the Acknowledgements and in the Funding section.

In the Acknowledgements,

“The authors would like to extend their sincere appreciation to the Researchers Supporting Project, King Saud University, Riyadh, Saudi Arabia for funding this work through the project number (RSP2024R197).”

now reads:

“The authors would like to extend their sincere appreciation to the Researchers Supporting Project, King Saud University, Riyadh, Saudi Arabia for funding this work through the project number (RSP2025R457).”

In the Funding section,

“This work is financially supported by the Researchers Supporting Project number (RSP2024R197), King Saud University, Riyadh, Saudi Arabia.”

now reads:

“This work is financially supported by the Researchers Supporting Project number (RSP2025R457), King Saud University, Riyadh, Saudi Arabia.”

The original Article has been corrected.

