# Supplementary material for: Structure based functional identification of an uncharacterized protein from Coxiella burnetii involved in adipogenesis
Source: Sci Rep. 2024 Jul 22;14:16789. doi: 10.1038/s41598-024-66072-3 (PMC11263603; doi:10.1038/s41598-024-66072-3)
Supplement: Supplementary file 1 — Supplementary Information. [file 41598_2024_66072_MOESM1_ESM.docx]

**Structure based functional identification of an uncharacterized protein from *Coxiella burnetii* involved in adipogenesis by use of in silico approach**

**Supplementary material**

Table 1. List of databases used for the in-silico characterization of hypothetical protein.

| S.L. | Web servers | URL | Purpose |
| --- | --- | --- | --- |
| 1 | ExPASy | http://web.expasy.org/protparam | Predict the physicochemical properties |
| 2 | CELLO | http://cello.life.nctu.edu.tw/ | Predict the subcellular localization of protein |
| 3 | PSORTb | https://www.psort.org/psortb/ |  |
| 4 | SOSUIGramN | https://harrier.nagahama-i-bio.ac.jp/sosui/sosuigramn/sosuigramn_submit.html |  |
| 5 | PSLpred | http://crdd.osdd.net/raghava/pslpred/ |  |
| 6 | CCTOP | https://cctop.ttk.hu/ |  |
| 7 | Pfam | http://pfam.xfam.org/ | Predict the function |
| 8 | NCBI | https://www.ncbi.nlm.nih.gov/Structure/cdd/wrpsb.cgi |  |
| 9 | I-TASSER | https://zhanggroup.org/I-TASSER/ |  |
| 10 | INTERPRO | https://www.ebi.ac.uk/interpro/ | Motif discovery |
| 11 | MOTIF | http://www.genome.jp/tools/motif/ |  |
| 12 | Clustal Omega | https://www.ebi.ac.uk/Tools/msa/clustalo/ | Multiple sequence alignment |
| 13 | SOPMA | https://npsa-prabi.ibcp.fr/cgi-bin/npsa_automat.pl?page=/NPSA/npsa_sopma.html | Predict the secondary structure |
| 14 | PSIPRED | http://bioinf.cs.ucl.ac.uk/psipred |  |
| 15 | Swiss Model | https://swissmodel.expasy.org/interactive | Predict the tertiary structure |
| 16 | YASARA | http://www.yasara.org/minimizationserver.htm | Energy minimization of the 3D model structure |
| 17 | PROCHECK | https://saves.mbi.ucla.edu/ | Ramachandran plot analysis |
| 18 | Verify3D | https://saves.mbi.ucla.edu/ | Structure validation |
| 19 | ERRAT | https://saves.mbi.ucla.edu/ |  |
| 20 | ProSA | https://prosa.services.came.sbg.ac.at/prosa.php | Protein structure analysis |
| 21 | CASTp | http://sts.bioe.uic.edu/castp/index.html | Active site predicts |
| 22 | STRING | https://string-db.org/ | Protein-protein interaction analysis |
| 23 | iMODS | http://imods.chaconlab.org/) | Molecular dynamic simulation |


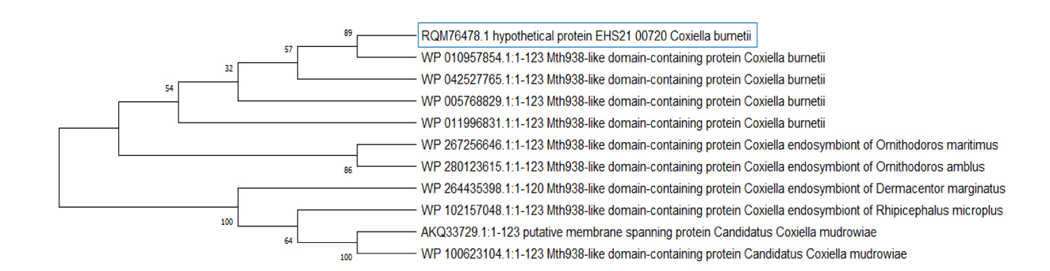


Figure 1. An evaluation of the uncharacterized protein's phylogenetic relationship to other proteins in the non-redundant database.


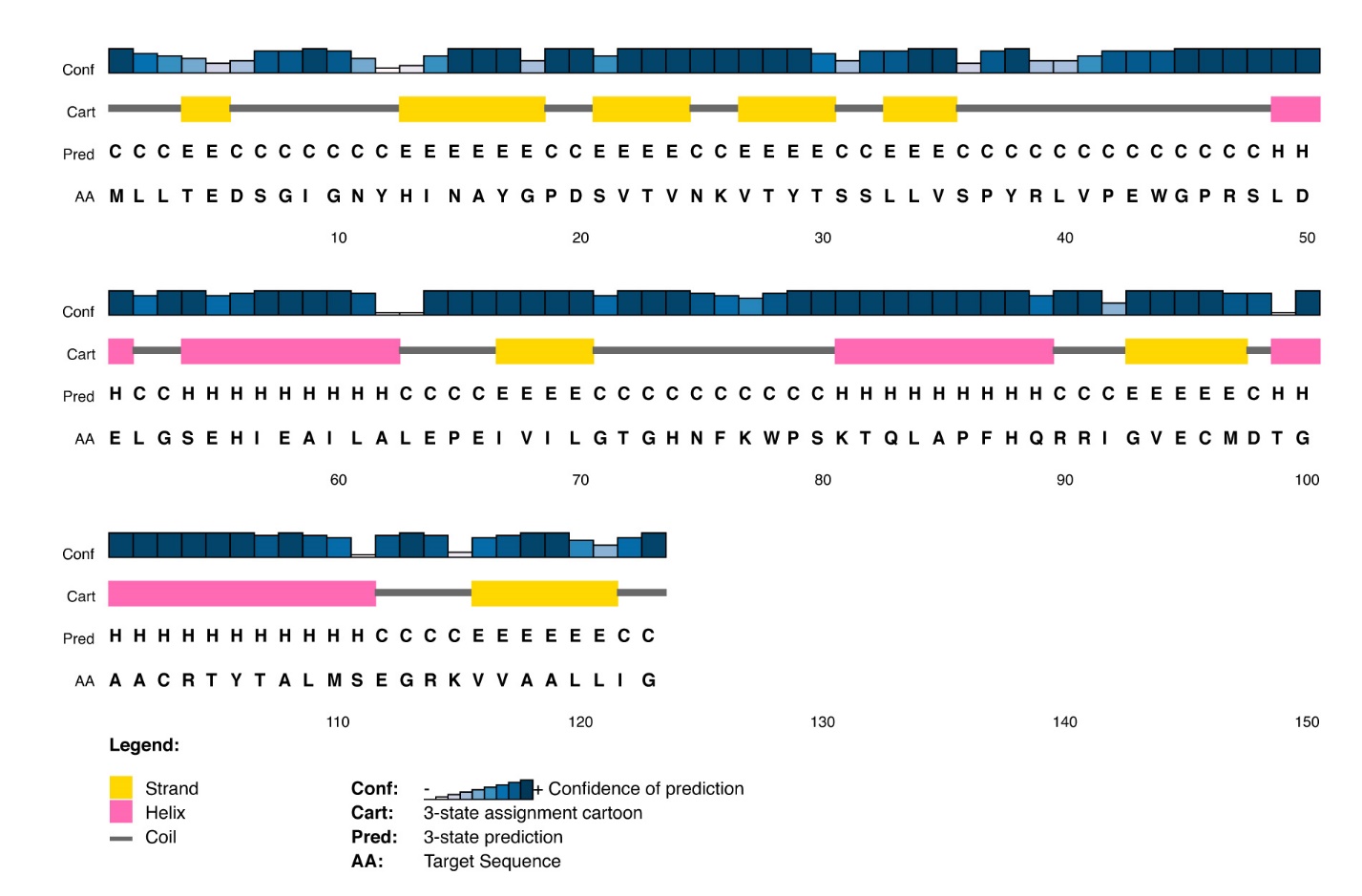


Figure 2. The PSIPRED server displays the predicted secondary structure of the protein along with a confidence score, providing an in-depth analysis of the structural characteristics expected based on the computational predictions.


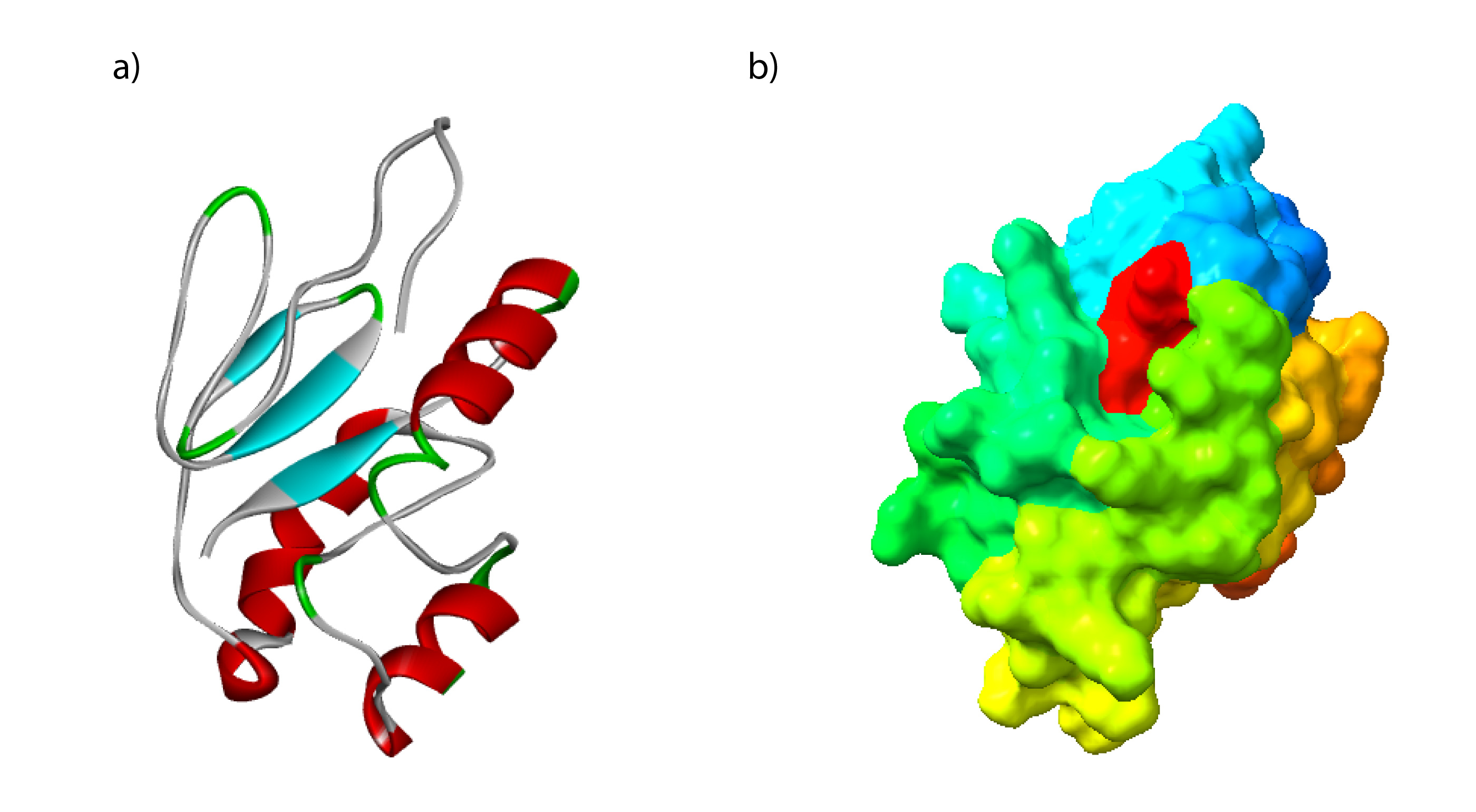


Figure 3. The predicted three-dimensional structure of the target protein is illustrated as follows: (a) a ribbon diagram of the protein, highlighting its folding patterns and organization, and (b) a surface diagram of the protein, showing its surface features and potential interaction sites.


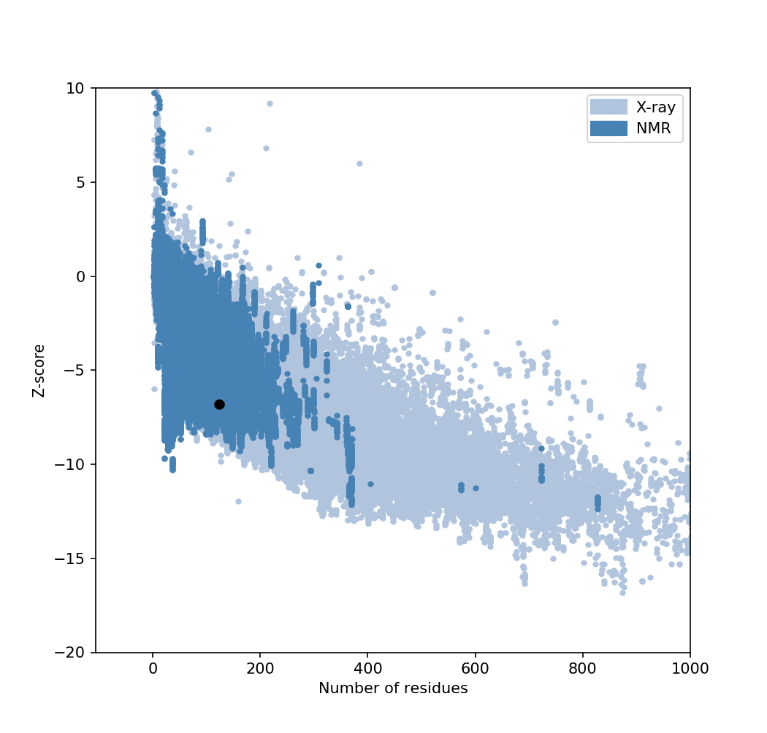


Figure 4. An evaluation of the anticipated structure's reliability, indicated by a Z score of -6.79.


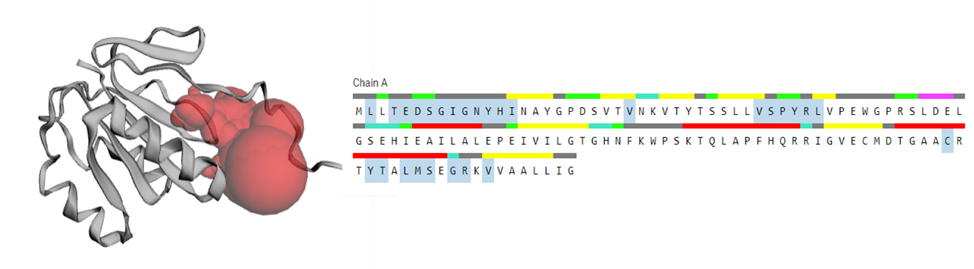


**Figure 5.** The CASTp server was utilized to identify the active site of the protein and its constituent amino acids.
